# Basal Forebrain Cholinergic System and Orexin Neurons: Effects on Attention

**DOI:** 10.3389/fnbeh.2017.00010

**Published:** 2017-01-31

**Authors:** Ines Villano, Antonietta Messina, Anna Valenzano, Fiorenzo Moscatelli, Teresa Esposito, Vincenzo Monda, Maria Esposito, Francesco Precenzano, Marco Carotenuto, Andrea Viggiano, Sergio Chieffi, Giuseppe Cibelli, Marcellino Monda, Giovanni Messina

**Affiliations:** ^1^Department of Experimental Medicine, Second University of NaplesNaples, Italy; ^2^Department of Clinical and Experimental Medicine, University of FoggiaFoggia, Italy; ^3^Department of Motor, Human and Health Science, University of Rome, “Foro Italico”Rome, Italy; ^4^Department of Mental Health, Physical and Preventive Medicine, Second University of NaplesNaples, Italy; ^5^Neapolitan Brain Group (NBG), Clinic of Child and Adolescent Neuropsychiatry, Department of Mental, Physical Health and Preventive Medicine, Second University of NaplesNaples, Italy; ^6^Department of Medicine, Surgery and Dentistry “Scuola Medica Salernitana”, University of SalernoSalerno, Italy

**Keywords:** attention, orexin, basal forebrain, lateral hypothalamus, acetylcholine

## Abstract

The basal forebrain (BF) cholinergic system has an important role in attentive functions. The cholinergic system can be activated by different inputs, and in particular, by orexin neurons, whose cell bodies are located within the postero-lateral hypothalamus. Recently the orexin-producing neurons have been proved to promote arousal and attention through their projections to the BF. The aim of this review article is to summarize the evidence showing that the orexin system contributes to attentional processing by an increase in cortical acetylcholine release and in cortical neurons activity.

## Introduction

Attention may be defined as the behavioral and cognitive process that allows us to select the information present in our environment on the basis of their relevance along with the ability to ignore irrelevant stimuli (Sarter et al., 2001). It consists of several components such as sustained, selective and divided attention, which are responsible for the control of the flow of information in the cognitive system (Rieger et al., 2003). Attention involves both top-down processes (knowledge-driven mechanisms) and bottom-up processes (mechanisms driven mainly by the characteristics of the target stimulus and its sensory context; Sarter et al., 2001; Chieffi et al., 2004, 2012). These two processes drive the attentive focus control (Gazzaniga et al., 2002). Attentional processing comprises some generalized states of arousal which refers to the state of physiological reactivity ranging from sleep to excitement or panic (Coull, 1998; Fadel and Burk, 2010).

Changes in arousal typically are deduced from brain activity data (EEG), whereas the study of attention is based on behavioral studies. Moruzzi and Magoun (1949) first demonstrated that cerebral activation is related to changes in EEG waves and has a brainstem origin. The discovery and localization of the brainstem reticular arousal system (RAS) was subsequently made by Moruzzi and Magoun (1949). The more evident arousal effect on EEG activity is the “desynchronization” phenomenon. It refers to the rapid shift from high-amplitude low-frequency EEG activity, typical of sleep, to low-amplitude high-frequency electroencephalographic activity, typical of wakefulness. EEG was the earliest measure used to systematically examine human brain cortical activity. After a long period of decline in clinical interest, EEG is now attracting increasing scientific and clinical interest. This resurgence is due to ongoing advances in signal processing and visualization that increase the spatial resolution of EEG imaging and exploit its ability to image quick transient cortical events and more precise regional changes in cortical activity. For the last few decades, scalp channel EEG data have been analyzed principally either in the time domain via ERP trial averaging, or in the frequency domain using FFT that estimate spectral power within a given frequency. Although phenomena and definitions may vary, EEG spectral power variations are typically dominated by distinct changes in power in few frequency bands. The standard terminology for these bands is: delta (<4 Hz), theta (4–7 Hz), alpha (8–12 Hz), beta (13–25 Hz; often split into beta-1/sensorimotor rhythm (SMR), 13–16 Hz, and beta-2, 17–25 Hz), and gamma (25–50 Hz or even higher frequency broadband activity extending to 200 Hz or greater). Cerebral activation is also detected during rapid eye movement (REM) sleep. Experimental evidence suggest that arousal systems work differently during the wake state and the REM sleep (Krueger et al., 2016). Arousal effects arise from the stimulation of the mesopontine cholinergic nuclei (Montplaisir, 1975; Jones and Webster, 1988) and the locus coeruleus (LC; Steriade and McCarley, 1990), which consist principally of noradrenergic neurons. Conversely, during REM the monoaminergic (noradrenergic and serotoninergic) neurons are silent (Hobson et al., 1975; McGinty and Harper, 1976). It is possible to distinguish brain mechanisms involved in attention from arousal, thanks to changes in task performance following manipulations known to affect attention (De Gangi and Porges, 1990; Schiff and Plum, 2000; Fadel and Burk, 2010). In this case, the interpretation of data resulting from these manipulations is primarily based on behavioral performance data (e.g., detection rates, false alarm rates, etc.) (Sarter and Bruno, 1999; Sarter et al., 2001). The relationship between arousal and attention is not simple. Attentional performance improves with a moderate increase of arousal but drops dramatically during high excitement state (Easterbrook, 1959). On the other hand, sustained attention reduces arousal and induces drowsiness (Babkoff et al., 1991).

Furthermore, physiological studies and data collected on patients suffering from injuries or neurological diseases provide a wealth of information on the neural mechanisms of attention processes. Thus, it is important to determine the brain networks mediating attention both to understand the neural mechanisms underlying these cognitive functions to expand knowledge on neurodevelopmental disorders characterized by impairments in attentional functions (Sarter et al., 2001; Esposito and Carotenuto, 2010, 2014; Carotenuto et al., 2016). Among these networks, the basal forebrain (BF) cholinergic system is considered as a major component of top-down processes in the mediation of attention, it is known to play a role in several aspects of attentional function (Fadel and Burk, 2010; Viggiano et al., 2014) and to be necessary for normal attentional performance (Sarter et al., 2001; Boschen et al., 2009). This system can be activated by different afferent inputs and can influence how attentional resources are allocated (Chieffi et al., 2009; Fadel and Burk, 2010). Among the various afferent inputs to the BF cholinergic projection system, the hypothalamus represents an important source of projections. The available data demonstrate that orexin neurons, whose cell bodies are present in the lateral hypothalamus, contribute substantially to these projections (Cullinan and Záborszky, 1991). Orexin neurons have widespread projections to a number of brain regions, including cholinergic BF structures. In the last decade, several studies have focused on specific neuronal pathways through which the orexin-producing neurons may promote not only arousal, but also attention. Their results suggest that the basal forebrain may be a key site through which these neurons act. In this article, we review the effects of orexin-producing neurons and their projection to the BF to support the hypothesis that orexin system may contribute to attentional processing through increased cortical-acetylcholine (Ach) release.

## The Cholinergic Basal Forebrain System

**Figure 1:**
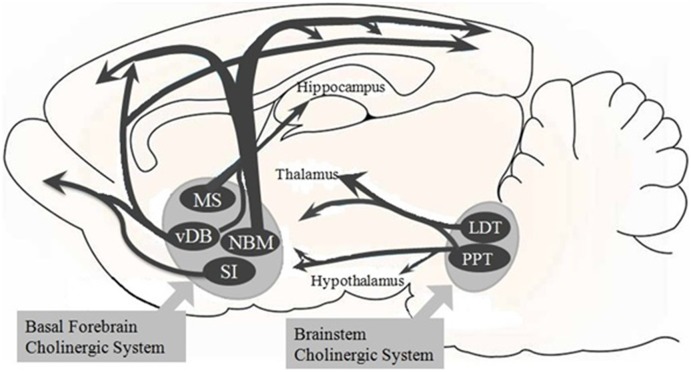
**Overview of the basal forebrain (BF) cholinergic pathway.** The BF cholinergic system of the Sprague-Dawley rats includes the medial septum (MS), vertical limbs of the diagonal band of Broca (vDB), nucleus basalis of Meynert (NBM), and substantia innominate (SI). The vDB and NBM have diffuse projections to all parts of the neocortex and to basolateral amygdala and olfactory bulb (these latter two are not shown here). The MS and vDB project to hippocampus. Besides, the brainstem cholinergic system projects to the thalamus and hypothalamus but also to the BF region. This system includes the pedunculopontine tegmental nucleus (PPT) and laterodorsal pontine tegmentum (LDT).

In the BF, cholinergic neurons are codistributed with several other cell populations, including GABAergic and various neurons containing calcium binding protein for example calbindin, calretinin or parvalbumin (Fadel and Burk, 2010). These neurons project to all areas and layers of the cortex (Sarter and Bruno, 1997). The cholinergic projections modulate the response of pyramidal cells to other cortical-glutamatergic inputs (McCormick, 1993), facilitating the bottom-up sensory information processing within the cortex (Figure 1; Muir et al., 1994; Sarter et al., 2001). Furthermore, the long radiating dendrites of the cholinergic BF neurons receive inputs from all the brainstem and hypothalamic arousal systems, for example cholinergic ponto-mesencephalic neurons, noradrenergic LC neurons, dopaminergic ventral-mesencephalic neurons, histaminergic tubero-mammillary neurons and orexinergic perifornical neurons (Jones and Cuello, 1989; Panula et al., 1989; Zaborszky and Cullinan, 1996; Peyron et al., 1998; Semba et al., 1998).

The cholinergic basal forebrain neurons have been implicated in mechanisms of synaptic plasticity, learning, memory, arousal and attention (McCormick, 1993; Leanza et al., 1996); all these functions are related to cortical activation (Jones, 2003). For instance, pharmacological manipulations of cholinergic receptors in extra-striate occipital and superior-medial parietal cortices affect attentional performance (Bentley et al., 2004), and lesions of the BF in monkeys also interfere with attention (Voytko et al., 1994).

The cholinergic basal forebrain neurons are hyperpolarized by ACh released by brainstem or forebrain neurons; both muscarinic and nicotinic receptors are involved in this effect, and could modulate the cortical and forebrain activity during particular states across the sleep–waking cycle (McCormick, 1993; Khateb et al., 1997). In the cerebral cortex and in the hippocampus, Ach release is maximal during wakefulness and REM sleep (Jasper and Tessier, 1971; Marrosu et al., 1995), while it decreases during non-REM sleep (Arrigoni et al., 2010). Inglis et al. (1994) suggested that the BF neurons may be involved, together with dopaminergic neurons, in the regulation of attention and in rewarding activities including food intake because high amount of Ach is released during eating. Many other neurotransmitters can excite the BF neurons, for example glutamate (Khateb et al., 1995a), noradrenaline (NA; Fort et al., 1995), histamine (Khateb et al., 1995a,b), orexin (Eggermann et al., 2001), or can inhibit them for example serotonin (Khateb et al., 1993).

## Overview of The Orexin Neurons

The orexin/hypocretins are neuropeptides synthesized by a cluster of neurons within the postero-lateral hypothalamus that produce excitatory effects on target neurons. Two independent research groups discovered simultaneously these neuropeptides in the late 1990s. One group named these peptides orexins, from the Greek word “orexis”, meaning “appetite”, because they seemed to be involved in the control of feeding and metabolism (Sakurai et al., 1998; Sakurai, 2007). The other group named these peptides hypocretins, because these peptides share significant sequence homology with the members of the glucagon/vasoactive intestinal polypeptide/secretin (incretin) family (de Lecea et al., 1998). Therefore, as hypocretin, de Lecea et al. (1998), intended to indicate a hypothalamic member of the incretin family. However, the terms are interchangeable in the literature. Orexin-A (orexin-A/hypocretin-1, Orx-A) and orexin-B (orexin-B/hypocretin-2, Orx -B) are cleaved from a single gene product, prepro-orexin (Sakurai et al., 1998). Orexins act on two different G-protein coupled receptors: orexin 1 receptor (Orx1R), which binds selectively Orx A, and orexin 2 receptor (Orx2R), which binds both Orx-A and Orx-B with equal affinity (Sakurai et al., 1998; Sakurai, 2007). Orexin neurons also release other neurotransmitters, such as glutamate on histamine tubero-mammillary neurons (critical for the maintenance of arousal), the inhibitory neuropeptide dynorphin (which also modulate appetite) and pentraxin (regulator of AMPA receptors clustering; Chou et al., 2001; Reti et al., 2002; Schone et al., 2012). The orexin neurons may integrate a variety of interoceptive and homeostatic signals related to environmental, physiological and emotional stimuli to promote wakefulness and behavioral arousal in response to emotions, stress, hunger and circadian rhythms (Yoshida et al., 2006; Viggiano et al., 2009). Furthermore several brain regions involved in the central regulation of autonomic and endocrine processes or attention are targets of extensive orexin projections (Horvath et al., 1999; Chieffi et al., 2014a,b). Neurons containing the neuropeptide orexin send axons to numerous regions, throughout the central nervous system; their projections are widely distributed in the brain (Chemelli et al., 1999). These neurons innervate all brain regions known to promote wakefulness and arousal (Saper et al., 2005) including the cerebral cortex, BF, tubero-mammillary nucleus (TMN), LC, and dorsal raphe (DR; Peyron et al., 1998; Yoshida et al., 2006). Furthermore, they innervate brain nuclei that regulate motivation and emotions (Sakurai and Mieda, 2011; Thompson and Borgland, 2011; Di Bernardo et al., 2014), and brain regions that regulate motor and autonomic functions (Nattie and Li, 2012). Thus, the orexin system is anatomically well positioned to coordinate many aspects of arousal and attention (Alexandre et al., 2013). Indeed, the orexin neurons are important in regulation of sleep/wakefulness states and lack of the peptide or the receptor caused narcolepsy in humans, dogs and mice (Chemelli et al., 1999; Lin et al., 1999; Thannickal et al., 2000).

## Orexin and Attention

In attention regulation, orexins play a significant role likely via interactions with multiple ascending neuromodulatory systems, including dopamine neurons in the ventral midbrain (Vittoz and Berridge, 2006), noradrenergic neurons in the LC (Horvath et al., 1999; Espana et al., 2005) and the basal forebrain cholinergic system (Fadel and Burk, 2010). In the BF, orexin peptides increase cell activity and Ach release, thus they modulate attentional mechanisms (Fadel and Burk, 2010). Attentional deficits present in neurodegenerative conditions such as Alzheimer’s disease, schizophrenia, drug addiction, and age-related cognitive decline may be related with alterations in the interactions between orexin neurons and cortical ACh neurons (Fadel and Burk, 2010). An imbalance in orexin regulation may also be involved in the pediatric Attention Deficit Hyperactivity Disorder syndrome (ADHD), comprising cognitive alterations, and in narcoleptic and/or obese and/or migrainous subjects as summarized in the Prader-Willi syndrome (Cortese et al., 2008; Carotenuto et al., 2009; Verrotti et al., 2013, 2015a,b; Morandi et al., 2015; Miano et al., 2016). Orexin neurons activity varies with the degree of arousal and is linked to heightened attentional states. Their activity promotes arousal, with maximal discharge during active wakefulness (Lee et al., 2005; Mileykovskiy et al., 2005; Viggiano et al., 2010), while their discharge decreases during quiet waking, in the absence of movement, and are silent in slow wave sleep and tonic periods of REM sleep, with occasional burst of activity during REM sleep (Lee et al., 2005; Mileykovskiy et al., 2005). In addition to arousal, orexins promote eating and are likely to have a role in physiological functions such as regulation of blood pressure, the neuroendocrine system, body temperature, and energy homeostasis (Peyron et al., 1998; Hara et al., 2001; Jones, 2003; Messina et al., 2014). Blouin et al. (2013) demonstrated that, in the human brain, Orx-A levels are maximal during positive emotion, social interaction, anger and increase at wake onset, suggesting that these levels are linked to specific emotions and state transitions.

## Network Regulation of Orexin Neurons

**Figure 2:**
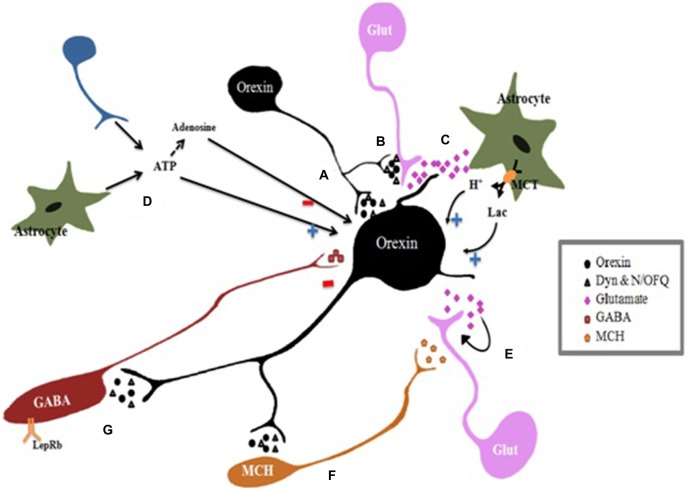
**Regulation of orexin neurons.** Orexin neurons activity is controlled by positive and negative feedback mechanisms mediated by neurotransmitters released by lateral hypothalamus/perifornical area (LH/PFA) neurons. Orexin neurons corelease excitatory neurotransmitters orexin and inhibitory transmitters dynorphin (Dyn) and nociceptin/orphanin FQ (N/OFQ). **(A)** Direct effect: all of neurotransmitters coreleased by Orexin neurons form a feedback which directly affects postsynaptic orexin neurons. **(B)** Synaptic modulation: orexins modulate presynaptic glutamate release at excitatory synapses. Besides, Dyn attenuates glutamate release acting on presynaptic excitatory terminals, while N/OFQ inhibits both excitatory and inhibitory transmission. The balance between the excitatory and inhibitory effects determines the activity levels of the postsynaptic cell. **(C)** Indirect effects: Regulation of orexin neurons by astrocytes: Glutamate activates astrocytes which release lactate (Lac) and protons (H+) into the extracellular space through monocarboxylate transporters (MCTs). Orexin neurons metabolize astrocyte-derived lactate as an energy substrate to sustain activity. Furthermore, extracellular pH decreases due to MCT activity resulting in depolarization of orexin neurons. **(D)** Adenosine triphosphate (ATP) effects: ATP released by astrocytes and neurons, stimulates orexin neurons depolarizing them through the ionotropic P2X receptors. Ectonucleotidases hydrolyze ATP releasing into adenosine in the extracellular space which inhibits orexin neurons. **(E)** Autoinhibition: the glutamate released synaptically creates a negative feedback loop acting on presynaptic autoreceptors to inhibit glutamate release. **(F)** Melanin concentrating hormone (MCH) neurons are directly depolarized by Orexin A and B which stimulate presynaptic glutamate release, whereas dynorphin and N/OFQ induce direct hyperpolarization of MCH neurons. **(G)** Leptin receptor-expressing GABAergic neurons are excited by leptin and use GABA as a neurotransmitter. Leptin inhibits indirectly orexin neurons by activating these inhibitory LepRb+ neurons. In summary, the balance between the excitatory and inhibitory effects determines the activity levels of the orexin neurons. Glut, glutamate; (+), stimulation; (−) inhibition.

Orexin neurons are controlled by positive and negative feedback mechanisms mediated by the lateral hypothalamus/perifornical area (LH/PFA; Figure 2). The Orx2R, Orx-A or Orx-B form a positive-feedback loop which opens nonselective cation channels and depolarizes orexin neurons and modulates presynaptic glutamate release (Yamanaka et al., 2010). Indirectly, glutamatergic transmissions stimulate orexin neurons through glutamate activation of astrocytes that release lactate and protons into the extracellular space through monocarboxylate transporters (MCTs; Pellerin et al., 1998; Burt et al., 2011). Furthermore, to sustain physical activity, orexin neurons metabolize astrocyte-derived lactate as an energy source; moreover, the release of protons due to MCT activity causes a local decrease in extracellular pH that can result in depolarization of orexin neurons (Williams et al., 2007). Even adenosine triphosphate (ATP), released by astrocytes and neurons, has an excitatory effect on orexin neurons through the ionotropic P2X receptors (Wollmann et al., 2005). ATP can be hydrolyzed by ectonucleotidases releasing adenosine in the extracellular space (Wall and Dale, 2008) which inhibits voltage-gated Ca^2+^ currents in orexin neurons leading to their inhibition (Liu and Gao, 2007). Negative feedback pathways have also been identified, for example Dynorphin and Nociceptin/Orphanin FQ (N/OFQ), either co-expressed by orexin neurons (Chou et al., 2001; Maolood and Meister, 2010). Dynorphin attenuates glutamate release acting on presynaptic excitatory terminals, while N/OFQ inhibits both excitatory and inhibitory transmission (Li and van den Pol, 2006). The balance between the excitatory and inhibitory effects determines the activity levels of the postsynaptic cell (Burt et al., 2011). In addition, the glutamate released synaptically creates a negative feedback loop acting on presynaptic autoreceptors to inhibit glutamate and GABA released through group III metabotropic glutamate receptors (mGluRs; Acuna-Goycolea et al., 2004). Even other distinct neuronal populations in the LH/PFA create synaptic contacts with orexin neurons for example neurons expressing melanin concentrating hormone (MCH) and leptin receptor-expressing (LepRb+) neurons. The MCH neurons form reciprocal connections with orexin neurons and are directly depolarized by Orexin A and B which stimulate presynaptic glutamate release, whereas dynorphin and N/OFQ directly induce hyperpolarization of MCH neurons (Li and van den Pol, 2006). MCH can reduce the presynaptic glutamate release induced by orexin receptors to antagonize the excitatory effects on orexin neurons (Rao et al., 2008). Leptin receptor-expressing (LepRb+) neurons are excited by leptin and use GABA as a neurotransmitter (Leinninger et al., 2009). Leptin inhibits orexin neurons through hyperpolarization of these neurons (Yamanaka et al., 2003), because the activation of the LepRb+ neurons produces an inhibition of orexin neurons (Burt et al., 2011). Orexin neurons are also innervated by afferents of non-cholinergic terminals from the BF cholinergic cell area. BF glutamatergic neurons can excite orexin neurons involved in arousal, whereas GABAergic neurons can inhibit orexin neurons promoting behavioral quiescence and sleep (Henny and Jones, 2006). In summary, different peptides released by orexin neurons or distinct populations of LH/PFA neurons modulate orexin neurons and exert different excitatory and inhibitory influences during wake or sleep states. Others studies showed that LC neurons have an important role in waking and REM sleep (REMS; Mallick et al., 2012; Choudhary et al., 2014). Kumar et al. (2012) have constructed a mathematical model of waking, NREMS and REMS, showning the importance of orexinergic neurons in stabilizing the wake-sleep cycle and demonstrating that even small changes in inputs to or from those neurons can have a large impact on the ensuing dynamics. The results from this model help to understand the neural mechanisms of regulation and the patho-physiology of REMS.

## Modulation of The Basal Forebrain Cholinergic System by Orexin Neurons: Effects on Attention

### Orexin Receptors in the Basal Forebrain

Orexin neurons have widespread projections to the basal forebrain that may promote arousal by activating the cortex. Orexin neurons also project onto BF cholinergic neurons and release orexins in the BF. Both Orx1R and Orx2R are expressed in the BF, and can activate cholinergic afferents (Marcus et al., 2001) and narcoleptic dogs lack OX2R (Lin et al., 1999). However, there are conflicting results from *in vitro* electrophysiological studies and BF orexin administration with regard to what type of orexin receptor subtypes are involved in the activation of cholinergic fibers. *in vitro* electrophysiological data indicate that both Orx-A and Orx-B can excite BF cholinergic cells, and that their effects are primarily Orx2R-mediated (Eggermann et al., 2001; Gotter et al., 2016). On the other hand, other studies suggested that the effects of orexin administration in the BF are primarily Orx1R-mediated (Espana et al., 2001). Using mice lacking orexin receptors, Alexandre et al. (2012) found that focal restoration of Orx1R and Orx2R in the substantia innominate (SI) partially rescued their ability to produce long bouts of wakefulness. Furthermore, Boschen et al. (2009) have blocked in rats the Orx1Rs through the administration of the Orx1R antagonist SB-334867 prior to a two-lever sustained attention task performance. Their results showed that Orx1R blockade decreased the accuracy in attention-demanding tasks and that some of these effects on attention may be mediated by BF corticopetal neurons. In summary, the two receptors may play different and complementary roles in response to varying types of homeostatic challenges (Fadel and Frederick-Duus, 2008).

### Orexin Activation of the Basal Forebrain

**Figure 3:**
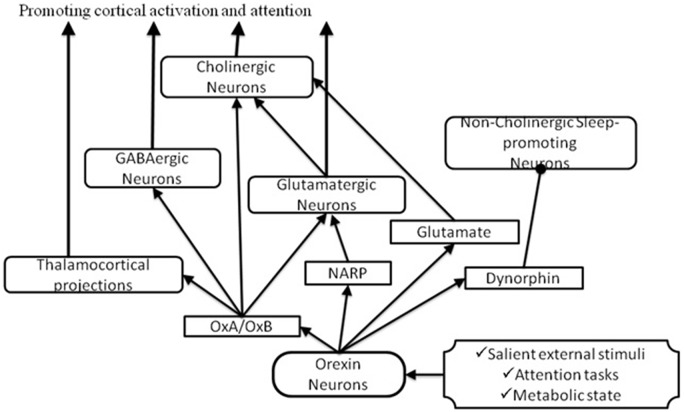
**Pathways through which orexin could activate the BF to promote attention.** In response to salient stimuli, orexin neurons produce several neuropeptides which promote cortical activation and attention by acting on cholinergic and non-cholinergic neurons. Arrows indicate excitatory inputs; dots indicate inhibitory inputs.

Different *in vitro* studies have tried to understand how orexin neurons activate the BF focusing primarily on the effects of orexins on medial septum (MS) neurons that project to the hippocampus and to the cortically projecting neurons of the BF. In the MS, orexins directly excite septo-hippocampal cholinergic neurons through the activation of the sodium, calcium exchanger and inhibition of potassium channels, presumably an inward rectifier, increasing hippocampal acetylcholine release and promoting arousal (Wu et al., 2004). Orx-A excites BF cholinergic neurons inducing cortical release of acetylcholine and increasing attention (see Figure 3; Arrigoni et al., 2010). Cortical acetylcholine levels increase even more under demanding attention tasks (Hasselmo and McGaughy, 2004) and orexin neurons increase firing to promote arousal and during exploratory behaviors in response to salient external stimuli (Mileykovskiy et al., 2005). It is also important to consider that local application of orexins to the BF promotes wakefulness and improves cognitive performance. In fact, the administration of orexins into the BF excites cholinergic neurons that release acetylcholine in the cerebral cortex and thereby promotes wakefulness (Eggermann et al., 2001; Espana et al., 2001; Fadel et al., 2005). Within the prefrontal cortex, orexins can also directly improve attentional processes relevant to executive aspects of attention. Lambe et al. (2005) demonstrated that infusions of Orx-B into the prefrontal cortex improved accuracy under high attentional demand by exciting the same thalamo-cortical synapses that are activated by acetylcholine from the BF. Thus, through an increased cortical acetylcholine release and a direct action on thalamo-cortical projections, orexins may promote cortical activation and attention. Orexin A can also modulate cholinergic neuron activity indirectly, because it increases local glutamate release within the basal forebrain. Indeed, Fadel and Frederick-Duus (2008) demonstrated that the administration of Orx-A in the BF increases local glutamate efflux. Furthermore, via an excitatory autoreceptor mechanism, Orx-A might increase BF glutamate release. Even non-cholinergic neurons of the BF may be excited by orexins, for example most of the GABAergic neurons of the BF (Fadel and Frederick-Duus, 2008; Arrigoni et al., 2010). In fact, orexin excites GABAergic neurons of the MS that project to the hippocampus (Wu et al., 2004) and GABAergic neurons of the magnocellular preoptic nucleus and substantia innominata (MCPO/SI; Blanco-Centurion et al., 2006). Blanco-Centurion et al. (2006) studied the relative contribution of non cholinergic neurons to arousal; they found that after selective lesion of the basal forebrain-cholinergic neurons in the rats., the microinjection of Orx-A into the BF increased waking and still promoted arousal; this finding indicates that cholinergic neurons are not essential for the effects of Orx-A and, thus, suggests that Orx-A acts also on non-cholinergic neurons (Blanco-Centurion et al., 2006). These findings suggest that orexins may contribute to attentional processing in the BF, not excluding, however, that other neural circuits outside the BF may contribute to these effects (Boschen et al., 2009).

### Orexin, Circulating Factors and Basal Forebrain

Orexin neurons are responsive to circulating factors related to metabolic state, for example low plasma glucose, and are activated by food deprivation (Cai et al., 1999). It has been suggested that these neurons can provide a crucial regulation of arousal level in response to signals of energy balance, such as blood glucose, leptin, and food intake (Yamanaka et al., 2003; Esposito et al., 2014; Messina et al., 2014). Furthermore, Frederick-Duus et al. (2007) demonstrated that the administration of the toxin orexin B–saporin (which produces loss of orexin neurons in the LH/PFA) in food-restricted rats lead them to be insensible to the cholinergic response to presentation of palatable food. Moreover, animals pretreated with the Orx1R antagonist, SB-334867, show an increased feeding latency demonstrating that Orx1R activity is required for an appetitive food stimulus to increase cortical Ach released. Fadel and Frederick-Duus suggest that orexins are necessary for the activation of BF cholinergic neurons in response to a food-related stimulus and they are important for biasing the allocation of attentional resources toward cues related to the physiological status (Fadel and Frederick-Duus, 2008).

### Influence of Other Orexin Neuron Neurotransmitters on the Basal Forebrain

To better understand how orexin-producing neurons promote cortical activation, some studies have focused also on the neuropeptide dynorphin, which is synthesized in orexin neurons and have specific effects on different classes of BF neurons. Cholinergic neurons do not respond to dynorphin but are directly excited by Orx-A, but there are two more populations of non-cholinergic BF neurons. One of these populations is excited by Orx-A and do not respond to dynorphin; the other population of non-cholinergic sleep-promoting neurons, is inhibited by dynorphin but do not respond to Orx-A (Arrigoni et al., 2010). Therefore, the co-release of orexins and dynorphin can activate a synergistic mechanism that excites cholinergic and non-cholinergic wake-active neurons and inhibits non-cholinergic sleep-active neurons promoting attention and improving cognitive performance. In addition to dynorphin, orexin neurons also co-release glutamate that acts synergistically to excite BF via presynaptic glutamatergic mechanisms (Arrigoni et al., 2010; Fadel and Burk, 2010). Orexin neurons express also the neuronal activity-regulated pentraxin (Narp) that is involved in clustering of glutamatergic α-amino-3-hydroxy-5-methyl-4-isoxazolepropionic acid (AMPA) receptors (Reti et al., 2002) and may potentiate pre- or post-synaptic responses to glutamate (Arrigoni et al., 2010). Despite the need of more studies to understand the role of dynorphin, glutamate and NARP, targeted deletion of orexin seem to have different functional deficits than those induced by selective ablation of orexin neurons (Chemelli et al., 1999; Hara et al., 2001; Reti et al., 2002) suggesting that other secreted signaling molecules expressed in these neurons are involved in their effects.

### Involvement of the Orexin-Basal Forebrain Interactions in Narcolepsy

Narcolepsy is a disease characterized by excessive daytime sleepiness, sleep paralysis, instability of sleep onset and REM periods, and cataplexy (Weinhold et al., 2014). Reduced orexinergic function, due to a reduction of orexin peptides, or orexin neurons, or orexin receptors, is assumed to be a major cause of narcolepsy, clearly demonstrated by post mortem studies (Arrigoni et al., 2010). Neuropsychological impairments have been found in narcoleptic patients, for example a reduced performance in attention-demanding tasks (Fulda and Schulz, 2001). Some studies suggest that narcoleptic patients show deficits in attention even during normal wakefulness periods (Rieger et al., 2003). Furthermore, BF degeneration is associated with canine narcolepsy suggesting that a postsynaptic degeneration and, in turn, impaired ACh-dependent cognitive function may be caused by a deficit in orexin stimulation (Siegel et al., 1999; Fadel and Frederick-Duus, 2008; Monda et al., 2014). In human narcolepsy there are deficits in selective processing of relevant stimuli. It is possible that these deficits are due to a reduction in BF cholinergic signaling to the cortex (Fadel and Burk, 2010). Weinhold et al. (2014) have investigated the effect of intranasal administration of Orx-A in narcoleptic patients with cataplexy on sleep behavior and cognitive functions. In the test of divided attention these patients showed enhanced performances after orexin-A administration, as indicated by their mean reaction time and fewer false reactions. Their results confirmed the role of Orx-A as a REM sleep stabilizing factor and provided functional evidence for the Orx-A effects on attention in narcolepsy with cataplexy (Weinhold et al., 2014). Other studies demonstrated that the intranasal administration of Orx-A in sleep-deprived rhesus monkey is able to relieve the cognitive deficits produced by the loss of sleep (Deadwyler et al., 2007). Moreover, it has been suggested that nasal Orx-A administration may be an effective approach to the treatment of orexin deficiency in narcolepsy (Peyron et al., 2000; Thannickal et al., 2000).

## Conclusion

Collectively, the available data strongly support the hypothesis that orexin stimulation of the BF is able to promote cortical activation and attention by acting on cholinergic and non-cholinergic neurons in response to salient stimuli. In fact, orexins excite cholinergic neurons, thus the increase in acetylcholine release within the cerebral cortex contributes to the cortical activation associated with attention. We have reviewed evidence suggesting that the BF may be a key target through which orexin neurons promote attention, even if many questions remain to be answered. Defining if orexin signaling in the BF is sufficient to maintain attention and the interaction between orexin and dynorphin within the BF should provide novel data to explain the role of orexin in several aspects of attention.

## Author Contributions

IV, AM, MC and AVa carried out the study; AVi, FM, TE, VM, ME and FP participated in the design of the study; SC, GC, MM and GM participated in the design and coordination and helped to draft the manuscript. All authors read and approved the final manuscript.

## Conflict of Interest Statement

The authors declare that the research was conducted in the absence of any commercial or financial relationships that could be construed as a potential conflict of interest.

## References

[B1] Acuna-Goycolea C. Li Y. van den Pol A. N. (2004). Group III metabotropic glutamate receptors maintain tonic inhibition of excitatory synaptic input to hypocretin/orexin neurons. J. Neurosci. 24, 3013–3022. 10.1523/JNEUROSCI.5416-03.2004 15044540 PMC6729849

[B2] Alexandre C. Andermann M. L. Scammell T. E. (2013). Control of arousal by the orexin neurons. Curr. Opin. Neurobiol. 23, 752–759. 10.1016/j.conb.2013.04.008 23683477 PMC3783629

[B3] Alexandre C. Mochizuki T. Arrigoni E. Yamamoto M. Clark E. Scammell T. E. (2012). Orexin signaling in the basal forebrain promotes EEG activation and wakefulness. Sleep 35:A31.

[B4] Arrigoni E. Mochizuki T. Scammell T. E. (2010). Activation of the basal forebrain by the orexin/hypocretin neurones. Acta Physiol. 198, 223–235. 10.1111/j.1748-1716.2009.02036.x 19723027 PMC2938067

[B5] Babkoff H. Caspy T. Mikulincer M. (1991). Subjective sleepiness ratings: the effects of sleep deprivation, circadian rhythmicity and cognitive performance. Sleep 14, 534–539. 1798887 10.1093/sleep/14.6.534

[B6] Bentley P. Husain M. Dolan R. J. (2004). Effects of cholinergic enhancement on visual stimulation, spatial attention and spatial working memory. Neuron 41, 969–982. 10.1016/s0896-6273(04)00145-x 15046728

[B28] Di Bernardo G. Messina G. Capasso S. Del Gaudio S. Cipollaro M. Peluso G.. (2014). Sera of overweight people promote *in vitro* adipocyte differentiation of bone marrow stromal cells. Stem Cell Res. Ther. 5:4. 10.1186/scrt393 24405848 PMC4055107

[B7] Blanco-Centurion C. A. Shiromani A. Winston E. Shiromani P. J. (2006). Effects of hypocretin-1 in 192-IgG-saporin-lesioned rats. Eur. J. Neurosci. 24, 2084–2088. 10.1111/j.1460-9568.2006.05074.x 17067305

[B8] Blouin A. M. Fried F. Wilson C. Staba R. J. Behnke E. J. Lam H. A.. (2013). Human hypocretin and melanin-concentrating hormone levels are linked to emotion and social interaction. Nat. Commun. 4:1547. 10.1038/ncomms2461 23462990 PMC3595130

[B9] Boschen K. E. Fadel J. R. Burk J. A. (2009). Systemic and intrabasalis administration of the orexin-1 receptor antagonist, SB-334867, disrupts attentional performance in rats. Psychopharmacology (Berl) 206, 205–213. 10.1007/s00213-009-1596-2 19575184

[B10] Burt J. Alberto C. O. Parsons M. P. Hirasawa M. (2011). Local network regulation of orexin neurons in the lateral hypothalamus. Am. J. Physiol. Regul. Integr. Comp. Physiol. 301, R572–R580. 10.1152/ajpregu.00674.2010 21697524

[B11] Cai X. J. Widdowson P. S. Harrold J. Wilson S. Buckingham R. E. Arch J. R.. (1999). Hypothalamic orexin expression: modulation by blood glucose and feeding. Diabetes 48, 2132–2137. 10.2337/diabetes.48.11.2132 10535445

[B12] Carotenuto M. Esposito M. Cortese S. Laino D. Verrotti A. (2016). Children with developmental dyslexia showed greater sleep disturbances than controls including problems initiating and maintaining sleep. Acta Paediatr. 105, 1079–1082. 10.1111/apa.13472 27173764

[B13] Carotenuto M. Santoro N. Grandone A. Santoro E. Pascotto C. Pascotto A.. (2009). The insulin gene variable number of tandemrepeats (INS VNTR) genotype and sleep disordered breathing in childhood obesity. J. Endocrinol. Invest. 32, 752–755. 10.1007/BF03346531 19574727

[B14] Chemelli R. M. Willie J. T. Sinton C. M. Elmquist J. K. Scammell T. Lee C.. (1999). Narcolepsy in orexin knockout mice: molecular genetics of sleep regulation. Cell 98, 437–451. 10.1016/S0092-8674(00)81973-X 10481909

[B15] Chieffi S. Conson M. Carlomagno S. (2004). Movement velocity effects on kinaesthetic localisation of spatial positions. Exp. Brain Res. 158, 421–426. 10.1007/s00221-004-1916-z 15127172

[B16] Chieffi S. Iachini T. Iavarone A. Messina G. Viggiano A. Monda M. (2014a). Flanker interference effects in a line bisection task. Exp. Brain Res. 232, 1327–1334. 10.1007/s00221-014-3851-y 24496492

[B17] Chieffi S. Iavarone A. Iaccarino L. La Marra M. Messina G. De Luca V.. (2014b). Age-related differences in distractor interference on line bisection. Exp. Brain Res. 232, 3659–3664. 10.1007/s00221-014-4056-0 25092273

[B18] Chieffi S. Iavarone A. Viggiano A. Monda M. Carlomagno S. (2012). Effect of a visual distractor on line bisection. Exp. Brain Res. 219, 489–498. 10.1007/s00221-012-3106-8 22576681

[B19] Chieffi S. Secchi C. Gentilucci M. (2009). Deictic word and gesture production: their interaction. Behav. Brain Res. 203, 200–206. 10.1016/j.bbr.2009.05.003 19433113

[B20] Chou T. C. Lee C. E. Lu J. Elmquist J. K. Hara J. Willie J. T.. (2001). Orexin (hypocretin) neurons contain dynorphin. J. Neurosci. 21:RC168. 11567079 10.1523/JNEUROSCI.21-19-j0003.2001 PMC6762880

[B21] Choudhary R. C. Khanday M. A. Mitra A. Mallick B. N. (2014). Perifornical orexinergic neurons modulate REM sleep by influencing locus coeruleus neurons in rats. Neuroscience 279, 33–43. 10.1016/j.neuroscience.2014.08.017 25168734

[B22] Cortese S. Konofal E. Lecendreux M. (2008). Alertness and feeding behaviors in ADHD: Does the hypocretin/orexin system play a role? Med. Hypotheses 71, 770–775. 10.1016/j.mehy.2008.06.017 18678446

[B23] Coull J. T. (1998). Neural correlates of attention and arousal: Insights from electrophysiology, functional neuroimaging and psychopharmacology. Prog. Neurobiol. 55, 343–361. 10.1016/s0301-0082(98)00011-2 9654384

[B24] Cullinan W. E. Záborszky L. (1991). Organization of ascending hypothalamic projections to the rostral forebrain with special reference to the innervation of cholinergic projection neurons. J. Comp. Neurol. 306, 631–667. 10.1002/cne.903060408 2071698

[B27] Deadwyler S. A. Porrino L. Siegel J. M. Hampson R. E. (2007). Systemic and nasal delivery of orexin-A (Hypocretin-1) reduces the effects of sleep deprivation on cognitive performance in nonhuman primates. J. Neurosci. 27, 14239–14247. 10.1523/JNEUROSCI.3878-07.2007 18160631 PMC6673447

[B29] Easterbrook J. A. (1959). The effect of emotion on cue utilization and the organization of behavior. Psychol. Rev. 66, 183–201. 10.1037/h0047707 13658305

[B30] Eggermann E. Serafin M. Bayer L. Machard D. Saint-Mleux B. Jones B. E.. (2001). Orexins/hypocretins excite basal forebrain cholinergic neurones. Neuroscience 108, 177–181. 10.1016/s0306-4522(01)00512-7 11734353

[B31] Espana R. A. Baldo B. A. Kelley A. E. Berridge C. W. (2001). Wake-promoting and sleep-suppressing actions of hypocretin (orexin): basal forebrain sites of action. Neuroscience 106, 699–715. 10.1016/s0306-4522(01)00319-0 11682157

[B32] Espana R. A. Reis K. M. Valentino R. J. Berridge C. W. (2005). Organization of hypocretin/orexin efferents to locus coeruleus and basal forebrain arousal-related structures. J. Comp. Neurol. 481, 160–178. 10.1002/cne.20369 15562511

[B33] Esposito M. Carotenuto M. (2010). Borderline intellectual functioning and sleep: the role of cyclic alternating pattern. Neurosci. Lett. 485, 89–93. 10.1016/j.neulet.2010.08.062 20813159

[B34] Esposito M. Carotenuto M. (2014). Intellectual disabilities and power spectra analysis during sleep: a new perspective on borderline intellectual functioning. J. Intellect. Disabil. Res. 58, 421–429. 10.1111/jir.12036 23517422

[B35] Esposito M. Serpe F. P. Diletti G. Messina G. Scortichini G. La Rocca C.. (2014). Serum levels of polychlorinated dibenzo-p-dioxins, polychlorinated dibenzofurans and polychlorinated biphenyls in a population living in the Naples area, southern Italy. Chemosphere 94, 62–69. 10.1016/j.chemosphere.2013.09.013 24112656

[B37] Fadel J. Burk J. A. (2010). Orexin/hypocretin modulation of the basal forebrain cholinergic system: role in attention. Brain Res. 131, 112–123. 10.1016/j.brainres.2009.08.046 19699722 PMC2819645

[B36] Fadel J. Frederick-Duus D. (2008). Orexin/hypocretin modulation of the basal forebrain cholinergic system: insights from *in vivo* microdialysis studies. Pharmacol. Biochem. Behav. 90, 156–162. 10.1016/j.pbb.2008.01.008 18281084

[B38] Fadel J. Pasumarthi R. Reznikov L. R. (2005). Stimulation of cortical acetylcholine release by orexin A. Neuroscience 130, 541–547. 10.1016/j.neuroscience.2004.09.050 15664710

[B39] Fort P. Khateb A. Pegna A. Muhlethaler M. Jones B. E. (1995). Noradrenergic modulation of cholinergic nucleus basalis neurons demonstrated by *in vitro* pharmacological and immunohistochemical evidence in the guinea pig brain. Eur. J. Neurosci. 7, 1502–1511. 10.1111/j.1460-9568.1995.tb01145.x 7551176

[B40] Frederick-Duus D. Guyton M. F. Fadel J. (2007). Food-elicited increases in cortical acetylcholine release require orexin transmission. Neuroscience 149, 499–507. 10.1016/j.neuroscience.2007.07.061 17928158

[B41] Fulda S. Schulz H. (2001). Cognitive dysfunction in sleep disorders. Sleep Med. Rev. 5, 423–445. 10.1053/smrv.2001.0157 12531152

[B25] De Gangi G. Porges S. (1990). Neuroscience Foundations of Human Performance. Rockville, MD: American Occupational Therapy Association Inc.

[B42] Gazzaniga M. S. Ivry R. B. Mangun G. R. (2002). Cognitive Neuroscience: The Biology of the Mind. 2nd Edn. New York, NY: W.W. Norton and Co.

[B43] Gotter A. L. Forman M. S. Harrell C. M. Stevens J. Svetnik V. Yee K. L.. (2016). Orexin 2 receptor antagonism is sufficient to promote NREM and REM sleep from mouse to man. Sci. Rep. 6:27147. 10.1038/srep27147 27256922 PMC4891657

[B44] Hara J. Beuckmann C. T. Nambu T. Willie J. T. Chemelli R. M. Sinton C. M.. (2001). Genetic ablation of orexin neurons in mice results in narcolepsy, hypophagia and obesity. Neuron 30, 345–354. 10.1016/S0896-6273(01)00293-8 11394998

[B45] Hasselmo M. E. McGaughy J. (2004). High acetylcholine levels set circuit dynamics for attention and encoding and low acetylcholine levels set dynamics for consolidation. Prog. Brain Res. 145, 207–231. 10.1016/s0079-6123(03)45015-2 14650918

[B46] Henny P. Jones B. E. (2006). Innervation of orexin/hypocretin neurons by GABAergic, glutamatergic or cholinergic basal forebrain terminals evidenced by immunostaining for presynaptic vesicular transporter and postsynaptic scaffolding proteins. J. Comp. Neurol. 499, 645–661. 10.1002/cne.21131 17029265 PMC2426825

[B47] Hobson J. A. McCarley R. W. Wyzinski P. W. (1975). Sleep cycle oscillation: reciprocal discharge by two brainstem neuronal groups. Science 189, 55–58. 10.1126/science.1094539 1094539

[B48] Horvath T. L. Peyron C. Diano S. Ivanov A. Aston-Jones G. Kilduff T. S.. (1999). Hypocretin (orexin) activation and synaptic innervation of the locus coeruleus noradrenergic system. J. Comp. Neurol. 415, 145–159. 10.1002/(sici)1096-9861(19991213)415:2<145::aid-cne1>3.3.co;2-u 10545156

[B49] Inglis F. M. Day J. C. Fibiger H. C. (1994). Enhanced acetylcholine release in hippocampus and cortex during the anticipation and consumption of a palatable meal. Neuroscience 62, 1049–1056. 10.1016/0306-4522(94)90342-5 7845585

[B50] Jasper H. H. Tessier J. (1971). Acetylcholine liberation from cerebral cortex during paradoxical (REM) sleep. Science 172, 601–602. 10.1126/science.172.3983.601 4324472

[B51] Jones B. E. (2003). Arousal systems. Front. Biosci. 8, s438–s451. 10.2741/1074 12700104

[B53] Jones B. E. Cuello A. C. (1989). Afferents to the basal forebrain cholinergic cell area from pontomesencephalic-catecholamine, serotonin and acetylcholine—neurons. Neuroscience 31, 37–61. 10.1016/0306-4522(89)90029-8 2475819

[B52] Jones B. E. Webster H. H. (1988). Neurotoxic lesions of the dorsolateral pontomesencephalic tegmentum-cholinergic cell area in the cat. I. Effects upon the cholinergic innervation of the brain. Brain Res. 451, 13–32. 10.1016/0006-8993(88)90745-7 3251579

[B54] Khateb A. Fort P. Alonso A. Jones B. E. Muhlethaler M. (1993). Pharmacological and immunohistochemical evidence for aserotonergic input to cholinergic nucleus basalis neurons. Eur. J. Neurosci. 5, 541–547. 10.1111/j.1460-9568.1993.tb00519.x 8261128

[B55] Khateb A. Fort P. Pegna A. Jones B. E. Muhlethaler M. (1995a). Cholinergic nucleus Basalis neurons are excited by histamine *in vitro*. Neuroscience 69, 495–506. 10.1016/0306-4522(95)00264-j 8552244

[B56] Khateb A. Fort P. Serafin M. Jones B. E. Muhlethaler M. (1995b). Rhythmical bursts induced by NMDA in cholinergic nucleus basalis neurones *in vitro*. J. Physiol. 487, 623–638. 10.1113/jphysiol.1995.sp020905 8544126 PMC1156650

[B57] Khateb A. Fort P. Williams S. Serafin M. Jones B. E. Mühlethaler M. (1997). Modulation of cholinergic nucleus basalis neurons by acetylcholine and N-methyl-D-aspartate. Neuroscience 81, 47–55. 10.1016/s0306-4522(97)00167-x 9300400

[B58] Krueger J. M. Frank M. G. Wisor J. P. Roy S. (2016). Sleep function: toward elucidating an enigma. Sleep Med Rev. 28, 46–54. 10.1016/j.smrv.2015.08.005 26447948 PMC4769986

[B59] Kumar R. Bose A. Mallick BN. (2012). A mathematical model towards understanding the mechanism of neuronal regulation of wake-NREMS-REMS states. PLoS One 7:e42059. 10.1371/journal.pone.0042059 22905114 PMC3414531

[B60] Lambe E. K. Olausson P. Horst N. K. Taylor J. R. Aghajanian G. K. (2005). Hypocretin and nicotine excite the same thalamocortical synapses in prefrontal cortex: correlation with improved attention in rat. J. Neurosci. 25, 5225–5229. 10.1523/JNEUROSCI.0719-05.2005 15917462 PMC6724823

[B61] Leanza G. Muir J. Nilsson O. G. Wiley R. G. Dunnett S. B. Bjorklund A. (1996). Selective immunolesioning of the basal forebrain cholinergic system disrupts short-term memory in rats. Eur. J. Neurosci. 8, 1535–1544. 10.1111/j.1460-9568.1996.tb01616.x 8758961

[B26] de Lecea L. Kilduff T. S. Peyron C. Gao X. B. Foye P. E. Danielson P. E.. (1998). The hypocretins: hypothalamus-specific peptides with neuroexcitatory activity. Proc. Natl. Acad. Sci. U S A 95, 322–327. 10.1073/pnas.95.1.322 9419374 PMC18213

[B62] Lee M. G. Hassani O. K. Jones B. E. (2005). Discharge of identified orexin/ hypocretin neurons across the sleep-waking cycle. J. Neurosci. 25, 6716–6720. 10.1523/JNEUROSCI.1887-05.2005 16014733 PMC6725432

[B63] Leinninger G. M. Jo Y. H. Leshan R. L. Louis G. W. Yang H. Barrera J. G.. (2009). Leptin acts via leptin receptor-expressing lateral hypothalamic neurons to modulate the mesolimbic dopamine system and suppress feeding. Cell Metab. 10, 89–98. 10.1016/j.cmet.2009.06.011 19656487 PMC2723060

[B65] Lin L. Faraco J. Li R. Kadotani H. Rogers W. Lin X.. (1999). The sleep disorder canine narcolepsy is caused by a mutation in the hypocretin (orexin) receptor 2 gene. Cell 98, 365–376. 10.1016/s0092-8674(00)81965-0 10458611

[B64] Li Y. van den Pol A. N. (2006). Differential target-dependent actions of coexpressed inhibitory dynorphin and excitatory hypocretin/orexin neuropeptides. J. Neurosci. 26, 13037–13047. 10.1523/JNEUROSCI.3380-06.2006 17167093 PMC6674960

[B66] Liu Z. W. Gao X. B. (2007). Adenosine inhibits activity of hypocretin/orexin neurons by the A1-receptor in the lateral hypothalamus: a possible sleep-promoting effect. J. Neurophysiol. 97, 837–848. 10.1152/jn.00873.2006 17093123 PMC1783688

[B67] Mallick B. N. Singh A. Khanday M. A. (2012). Activation of inactivation process initiates rapid eye movement sleep. Prog. Neurobiol. 97, 259–276. 10.1016/j.pneurobio.2012.04.001 22521402

[B68] Maolood N. Meister B. (2010). Nociceptin/orphanin FQ peptide in hypothalamic neurones associated with the control of feeding behaviour. J. Neuroendocrinol. 22, 75–82. 10.1111/j.1365-2826.2009.01946.x 20025627

[B69] Marcus J. N. Aschkenasi C. J. Lee C. E. Chemelli R. M. Saper C. B. Yanagisawa M.. (2001). Differential expression of orexin receptors 1 and 2 in the rat brain. J. Comp. Neurol. 435, 6–25. 10.1002/cne.1190 11370008

[B70] Marrosu F. Portas C. Mascia S. Casu M. A. Fà M. Giagheddu M.. (1995). Microdialysis measurement of cortical and hippocampal acetylcholine release during sleep-wake cycle in freely moving cats. Brain Res. 671, 329–332. 10.1016/0006-8993(94)01399-3 7743225

[B71] McCormick D. A. (1993). Actions of acetylcholine in the cerebral cortex and thalamus and implications for function. Prog. Brain Res. 98, 303–308. 10.1016/s0079-6123(08)62412-7 8248519

[B72] McGinty D. J. Harper R. M. (1976). Dorsal raphe neurons: depression of firing during sleep in cats. Brain Res. 101, 569–575. 10.1016/0006-8993(76)90480-7 1244990

[B73] Messina G. Dalia C. Tafuri D. Monda V. Palmieri F. Dato A.. (2014). Orexin-A controls sympathetic activity and eating behavior. Front. Psychol. 5:997. 10.3389/fpsyg.2014.00997 25250003 PMC4157463

[B74] Miano S. Esposito M. Foderaro G. Ramelli G. P. Pezzoli V. Manconi M. (2016). Sleep-related disorders in children with attention-deficit hyperactivity disorder: preliminary results of a full sleep assessment study. CNS Neurosci. Ther. 22, 906–914. 10.1111/cns.12573 27255788 PMC6492878

[B75] Mileykovskiy B. Y. Kiyashchenko L. I. Siegel J. M. (2005). Behavioral correlates of activity in identified hypocretin/orexin neurons. Neuron 46, 787–798. 10.1016/j.neuron.2005.04.035 15924864 PMC8281334

[B76] Monda M. Messina G. Scognamiglio I. Lombardi A. Martin G. A. Sperlongano P.. (2014). Short-term diet and moderate exercise in young overweight men modulate cardiocyte and hepatocarcinoma survival by oxidative stress. Oxid. Med. Cell. Longev. 2014:131024. 10.1155/2014/131024 25197428 PMC4147268

[B77] Montplaisir J. Y. (1975). Cholinergic mechanisms involve din cortical activation during arousal. Electroencephalogr. Clin. Neurophysiol. 38, 263–272. 10.1016/0013-4694(75)90247-3 46804

[B78] Morandi A. Bonnefond A. Lobbens S. Carotenuto M. Del Giudice E. M. Froguel P.. (2015). A girl with incomplete Prader-Willi syndrome and negative MS-PCR, found to have mosaic maternal UPD-15 at SNP array. Am. J. Med. Genet. A 167A, 2720–2726. 10.1002/ajmg.a.37222 26109092

[B79] Moruzzi G. Magoun H. W. (1949). Brain stem reticular formation and activation of the EEG. EEG Clin. Neurophysiol. 1, 455–473. 10.1016/0013-4694(49)90066-8 18421835

[B80] Muir J. L. Everitt B. J. Robbins T. W. (1994). AMPA-induced excitotoxic lesions of the basal forebrain: a significant role for the cortical cholinergic system in attentional function. J. Neurosci. 4, 2313–2326. 7512637 10.1523/JNEUROSCI.14-04-02313.1994 PMC6577145

[B81] Nattie E. Li A. (2012). Respiration and autonomic regulation and orexin. Prog. Brain Res. 198, 25–46. 10.1016/b978-0-444-59489-1.00004-5 22813968 PMC4405125

[B82] Panula P. Pirvola U. Auvinen S. Airaksinen M. S. (1989). Histamine-immunoreactive nerve fibers in the rat brain. Neuroscience 28, 585–610. 10.1016/0306-4522(89)90007-9 2710333

[B83] Pellerin L. Pellegri G. Bittar P. G. Charnay Y. Bouras C. Martin J. L.. (1998). Evidence supporting the existence of an activity-dependent astrocyte-neuron lactate shuttle. Dev. Neurosci. 20, 291–299. 10.1159/000017324 9778565

[B84] Peyron C. Faraco J. Rogers W. Ripley B. Overeem S. Charnay Y.. (2000). A mutation in a case of early onset narcolepsy and a generalized absence of hypocretin peptides in human narcoleptic brains. Nat. Med. 6, 991–997. 10.1038/79690 10973318

[B85] Peyron C. Tighe D. K. van den Pol A. N. de Lecea L. Heller H. C. Sutcliffe J. G.. (1998). Neurons containing hypocretin (orexin) project to multiple neuronal systems. J. Neurosci. 18, 9996–10015. 9822755 10.1523/JNEUROSCI.18-23-09996.1998 PMC6793310

[B86] Rao Y. Lu M. Ge F. Marsh D. J. Qian S. Wang A. H.. (2008). Regulation of synaptic efficacy in hypocretin/orexin-containing neurons by melanin concentrating hormone in the lateral hypothalamus. J. Neurosci. 28, 9101–9110. 10.1523/JNEUROSCI.1766-08.2008 18784290 PMC2562258

[B87] Reti I. M. Reddy R. Worley P. F. Baraban J. M. (2002). Selective expression of Narp, a secreted neuronal pentraxin, in orexin neurons. J. Neurochem. 82, 1561–1565. 10.1046/j.1471-4159.2002.01141.x 12354306

[B88] Rieger M. Mayer G. Gauggel S. (2003). Attention deficits in patients with narcolepsy. Sleep 26, 36–43. 12627730

[B89] Sakurai T. (2007). The neural circuit of orexin (hypocretin): maintaining sleep and wakefulness. Nat. Rev. Neurosci. 8, 171–181. 10.1038/nrn2092 17299454

[B90] Sakurai T. Amemiya A. Ishii M. Matsuzaki I. Chemelli R. M. Tanaka H.. (1998). Orexins and orexin receptors: a family of hypothalamic neuropeptides and G protein-coupled receptors that regulate feeding behavior. Cell 92, 573–585. 10.1016/s0092-8674(00)80949-6 9491897

[B91] Sakurai T. Mieda M. (2011). Connectomics of orexin-producing neurons: interface of systems of emotion, energy homeostasis and arousal. Trends Pharmacol. Sci. 32, 451–462. 10.1016/j.tips.2011.03.007 21565412

[B92] Saper C. B. Scammell T. E. Lu J. (2005). Hypothalamic regulation of sleep and circadian rhythms. Nature 437, 1257–1263. 10.1038/nature04284 16251950

[B93] Sarter M. Bruno J. P. (1997). Cognitive functions of cortical acetylcholine toward a unifying hypothesis. Brain Res. Rev. 23, 28–46. 10.1016/s0165-0173(96)00009-4 9063585

[B94] Sarter M. Bruno J. P. (1999). Cortical cholinergic inputs mediating arousal, attentional processing and dreaming: differential afferent regulation of the basal forebrain by telencephalic and brainstem afferents. Neuroscience 95, 933–952. 10.1016/s0306-4522(99)00487-x 10682701

[B95] Sarter M. Givens B. Bruno J. P. (2001). The cognitive neuroscience of sustained attention: where top-down meets bottom-up. Brain Res. Rev. 35, 146–160. 10.1016/s0165-0173(01)00044-3 11336780

[B96] Schiff N. D. Plum F. (2000). The role of arousal and “gating” systems in the neurology of impaired consciousness. J. Clin. Neurophysiol. 17, 438–452. 10.1097/00004691-200009000-00002 11085547

[B97] Schone C. Cao Z. F. Apergis-Schoute J. Adamantidis A. Sakurai T. Burdakov D. (2012). Optogenetic probing of fast glutamatergic transmission from hypocretin/orexin to histamine neurons *in situ*. J. Neurosci. 32, 12437–12443. 10.1523/JNEUROSCI.0706-12.2012 22956835 PMC6621251

[B300] Semba J. Mataki C. Yamada S. Nankai M. Toru M. (1998). Antidepressantlike effects of chronic nicotine on learned helplessness paradigm in rats. Biol. Psychiatry 43, 389–391. 10.1016/s0006-3223(97)00477-0 9513755

[B98] Siegel J. M. Nienhuis R. Gulyani S. Ouyang S. Wu M. F. Mignot E.. (1999). Neuronal degeneration in canine narcolepsy. J. Neurosci. 19, 248–257. 9870955 10.1523/JNEUROSCI.19-01-00248.1999 PMC6782381

[B99] Steriade M. McCarley R. W. (1990). Brainstem Control of Wakefulness and Sleep. New York, NY: Plenum Press.

[B100] Thannickal T. C. Moore R. Y. Nienhuis R. Ramanathan L. Gulyani S. Aldrich M.. (2000). Reduced number of hypocretin neurons in human narcolepsy. Neuron 27, 469–474. 10.1016/s0896-6273(00)00058-1 11055430 PMC8760623

[B101] Thompson J. L. Borgland S. L. (2011). A role for hypocretin/orexin in motivation. Behav. Brain Res. 217, 446–453. 10.1016/j.bbr.2010.09.028 20920531

[B102] Verrotti A. Agostinelli S. D’Egidio C. Di Fonzo A. Carotenuto M. Parisi P.. (2013). Impact of a weight loss program on migraine in obese adolescents. Eur. J. Neurol. 20, 394–397. 10.1111/j.1468-1331.2012.03771.x 22642299

[B103] Verrotti A. Carotenuto M. Altieri L. Parisi P. Tozzi E. Belcastro V.. (2015a). Migraine and obesity: metabolic parameters and response to a weight loss programme. Pediatr. Obes. 10, 220–225. 10.1111/ijpo.245 24990114

[B104] Verrotti A. Cusmai R. Laino D. Carotenuto M. Esposito M. Falsaperla R.. (2015b). Spalice A. Long-term outcome of epilepsy in patients with Prader-Willi syndrome. J. Neurol. 262, 116–123. 10.1007/s00415-014-7542-1 25326049

[B105] Viggiano A. Chieffi S. Tafuri D. Messina G. Monda M. De Luca B. (2014). Laterality of a second player position affects lateral deviation of basketball shooting. J. Sports Sci. 32, 46–52. 10.1080/02640414.2013.805236 23876006

[B106] Viggiano A. Nicodemo U. Viggiano E. Messina G. Monda M. De Luca B. (2010). Mastication overload causes an increase in O_2_− production into the subnucleus oralis of the spinal trigeminal nucleus. Neuroscience 166, 416–421. 10.1016/j.neuroscience.2009.12.071 20045451

[B107] Viggiano A. Vicidomini C. Monda M. Carleo D. Carleo R. Messina G.. (2009). Fast and low-cost analysis of heart rate variability reveals vegetative alterations in noncomplicated diabetic patients. J. Diabetes Complicat. 23, 119–123. 10.1016/j.jdiacomp.2007.11.009 18413209

[B108] Vittoz N. M. Berridge C. W. (2006). Hypocretin/orexin selectively increases dopamine efflux within the prefrontal cortex: involvement of the ventral tegmental area. Neuropsychopharmacology 31, 384–395. 10.1038/sj.npp.1300807 15988471

[B109] Voytko M. L. Olton D. S. Richardson R. T. Gorman L. K. Tobin J. R. Price D. L. (1994). Basal forebrain lesions in monkeys disrupt attention but not learning and memory. J. Neurosci. 14, 167–186. 8283232 10.1523/JNEUROSCI.14-01-00167.1994 PMC6576852

[B110] Wall M. Dale N. (2008). Activity-dependent release of adenosine: a critical re-evaluation of mechanism. Curr. Neuropharmacol. 6, 329–337. 10.2174/157015908787386087 19587854 PMC2701281

[B111] Weinhold S. L. Seeck-Hirschner M. Nowak A. Hallschmid M. Göder R. Baier P. C. (2014). The effect of intranasal orexin-A (hypocretin-1) on sleep, wakefulness and attention in narcolepsy with cataplexy. Behav. Brain Res. 262, 8–13. 10.1016/j.bbr.2013.12.045 24406723

[B112] Williams R. H. Jensen L. T. Verkhratsky A. Fugger L. Burdakov D. (2007). Control of hypothalamic orexin neurons by acid and CO_2_. Proc. Natl. Acad. Sci. U S A 104, 10685–10690. 10.1073/pnas.0702676104 17563364 PMC1965573

[B113] Wollmann G. Acuna-Goycolea C. van den Pol A. N. (2005). Direct excitation of hypocretin/orexin cells by extracellular ATP at P2X receptors. J. Neurophysiol. 94, 2195–2206. 10.1152/jn.00035.2005 15958604

[B114] Wu M. Zaborszky L. Hajszan T. van den Pol A. N. Alreja M. (2004). Hypocretin/orexin innervation and excitation of identified septohippocampal cholinergic neurons. J. Neurosci. 24, 3527–3536. 10.1523/JNEUROSCI.5364-03.2004 15071100 PMC6729747

[B115] Yamanaka A. Beuckmann C. T. Willie J. T. Hara J. Tsujino N. Mieda M.. (2003). Hypothalamic orexin neurons regulate arousal according to energy balance in mice. Neuron 38, 701–713. 10.1016/s0896-6273(03)00331-3 12797956

[B116] Yamanaka A. Tabuchi S. Tsunematsu T. Fukazawa Y. Tominaga M. (2010). Orexin directly excites orexin neurons through orexin 2 receptor. J. Neurosci. 30, 12642–12652. 10.1523/JNEUROSCI.2120-10.2010 20861370 PMC6633594

[B117] Yoshida K. McCormack S. España R. A. Crocker A. Scammell T. E. (2006). Afferents to the orexin neurons of the rat brain. J. Comp. Neurol. 494, 845–861. 10.1002/cne.20859 16374809 PMC2259441

[B118] Zaborszky L. Cullinan W. E. (1996). Direct catecholaminergic-cholinergic interactions in the basal forebrain. I. Dopamine-betahydroxylase and tyrosine hydroxylase input to cholinergic neurons. J. Comp. Neurol. 374, 535–554. 10.1002/(SICI)1096-9861(19961028)374:4<535::AID-CNE5>3.3.CO;2-0 8910734

